# Giant and reversible room-temperature elastocaloric effect in a single-crystalline Ni-Fe-Ga magnetic shape memory alloy

**DOI:** 10.1038/srep25500

**Published:** 2016-05-03

**Authors:** Yang Li, Dewei Zhao, Jian Liu

**Affiliations:** 1 Key Laboratory of Magnetic Materials and Devices, Ningbo Institute of Material Technology and Engineering, CAS, Ningbo 315201, China; 2 Zhejiang Province Key Laboratory of Magnetic Materials and Application Technology, Ningbo Institute of Material Technology and Engineering, CAS, Ningbo 315201, China; 3 School of Materials Science and Engineering, Shanghai University, Shanghai 200072, China

## Abstract

Good mechanical properties and large adiabatic temperature change render Heusler-type Ni_2_FeGa-based magnetic shape memory alloys as a promising candidate material for solid-state mechanical cooling application at ambient conditions. Superelastic behavior and associated elastocaloric effect strongly reply on deformation conditions (e.g. applied strain rate and strain level) of stress-induced martensitic transformations. With the aim of developing high-performance elastic cooling materials, in this work, we have carried out a systematic study on a Ni_54_Fe_19_Ga_27_ [420]-oriented single crystal by exploring the interaction between dynamic deformation parameters and thermal response. A giant and reversible adiabatic temperature change of ±7.5 K triggered by a low stress of 30 MPa was achieved. Such a high specific cooling performance thus offers the great advantage for the small scale solid-state mechanical cooling applications. Besides, a significant temporary residual strain effect has been observed at high strain rate, which is unfavorable for reversible elastocaloric effect but can be overcome by reducing stress hysteresis, and/or by elevating initial environmental temperature. The established criterion for the desirable reversible elastocaloric properties goes beyond the present system, and can be applicable for other shape memory alloys used for elastic cooling techniques.

Due to the severe energy crisis and environment issues nowadays, solid-state refrigeration technologies have attracted much attention as promising alternatives to replace the conventional vapor compression cooling. Associated multi-caloric phenomena, namely a reversible entropy/temperature change in response to the application of varied stimuli such as magnetic, mechanical and electric fields have been demonstrated in magnetocaloric, elastocaloric and electrocaloric materials^1^. Among these caloric effects, elastocaloric effect (eCE) has been considered as one of the most important candidates for non-vapor compression refrigeration system in the future^2^.

The elastocaloric materials undergo reversible stress-induced martensitic transformation and superelasticity, accompanied by drastic latent heat releasing and absorbing^3^. Such considerable thermal energy can be utilized for active regeneration. This opens a new window for shape memory alloys (SMAs) in the use of solid-state refrigeration. Some elastocaloric materials have exhibited larger reversible adiabatic temperature change ∆*T* and higher refrigerating capacity than magnetocaloric materials. In addition, TiNi and FePd shape memory elastocaloric alloys have better machinability to be fabricated into the optical geometries. These characters are of crucial importance for the design and implement of high performance solid-state cooling systems.

In the 19th century, eCE was first found in Indian rubber that warmed up when rapidly stretched^4^. The polymeric chains of nature rubber become oriented under uniaxial applied stress and the degree of order increases in the system, causing the decreased entropy in system^5^. In recent years, the large latent heat and related temperature changes have been reported in elastocaloric SMAs, such as Cu-Zn-Al alloys^6^,^7^, Ni-Mn-based alloys^8^,^9^, single-crystalline Fe-Pd^10^, Ni-Ti wires^3^ and films^11^. In these SMAs, eCE originates from the large entropy change of lattice vibration or lattice softening (e.g. Fe-Pd) during martensitic transformation (MT) from austenite to martensite. As a first-order diffusionless structural transformation, MT can be induced by changing temperature or applying uniaxial stress. When SMAs is heated or the applied stress is removed, a reverse MT takes place. Since the thermoelastic MT is always accompanied with the heat absorption and release, the notable temperature change can be detected when stress cycles around transformation temperatures. It is expected to obtain the large reversible temperature change by increasing the strain rate and transformation strain^12^. However, very high strain rate has negative impact on eCE as it is prone to bring about plastic deformation and therefore damage the fatigue life^13^. For the practical application, the main drawback of elastocaloric materials is its poor fatigue resistance under repeated thermal and/or stress cycling^3^. In order to prolong fatigue life, several approaches can be adopted. First, structural compatibilities can be realized by reducing the transformation stress layers. It has been proven that the doping of Cu in Ni-Ti significantly leads to a better fatigue life and more stabilized superelastic behavior^14^. Another strategy for saving fatigue life is the exploring of weak first-order transition with lattice softening^15^. Also, the fatigue resistance can be improved by changing deformation mode. For instance, by applying compression instead of tension, cracks will have little chance to grow and a much longer life span can be expected even for a material with relatively large cracks^3^. More importantly, it should be noted that transformation strain and strain rate on a given material have striking influences on fatigue life and functional stability, due to the resulting increased hysteresis and residual strain^13^. The stress hysteresis originates from the intrinsic mechanical dissipative heat of internal friction, when interface between austenite and martensite moves during transformation^16^. That is to say, the lower stress hysteresis is, the higher refrigeration efficiency would be. Therefore, there required crucial factors including large adiabatic temperature change, low hysteresis and good fatigue life in a realistic elastocaloric cooling system. The main goal of the present work is to simultaneously achieve the large reversible adiabatic temperature and reduced hysteresis in a Ni_54_Fe_19_Ga_27_ single crystal by exploring suitable dynamic deformation conditions.

As magnetically controlled actuator materials, Ni-Fe-Ga-based ferromagnetic shape memory alloys have exhibited large magnetic-field-induced strain and excellent shape memory properties^17^,^18^. Although a high magnetic field is required to drive the magnetostructural transformation in this system^19^, the martensitic transformation can be much more easily triggered by the application of uniaxial stress below 100 MPa^20^. In addition, the magnetic entropy change in Ni-Fe-Ga has the same sign with the structure item, which positively contributes to the total entropy change. In this context, we have recently observed a significant cooling effect of −9 K upon unloading in [001]-oriented Ni_50_Fe_19_Ga_27_Co_4_ single crystal above 350 K^21^ and a relatively lower ∆*T* of ±4 K during loading/unloading cycles in dual-phase Ni_54_Fe_19_Ga_27_ polycrystal at 298 K^20^. However, there is a need to develop new generation of elastocaloric materials capable of highly efficient cooling (large and reversible ∆*T*, small hysteresis and low driving force) around room temperature. In this work, by successfully optimizing transformation strain and strain rate, we have demonstrated a maximum reversible elastocaloric temperature change (∆*T*_loading_ = ∆*T*_unloading_) of ±7.5 K at 3.8% transformation strain under 30 MPa critical stress in a [420]-oriented Ni_54_Fe_19_Ga_27_ single crystal at 293 K.

## Results and Discussions

**Figure 1:**
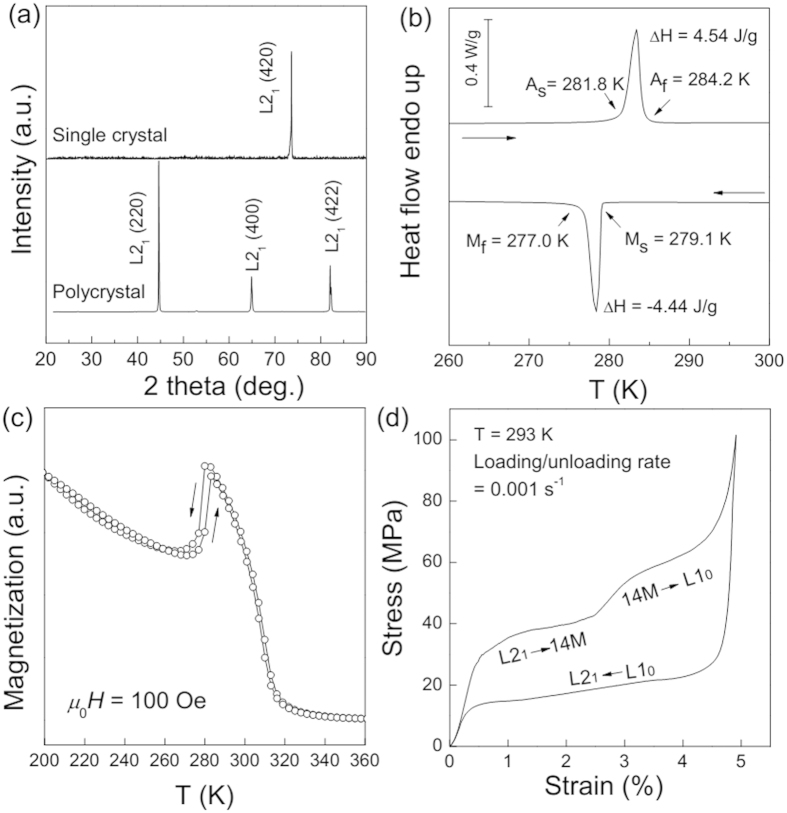
(**a**) XRD patterns of Ni_54_Fe_19_Ga_27_ single crystal and polycrystalline sample at room temperature. (**b**) DSC curve of the Ni_54_Fe_19_Ga_27_ single crystal. (**c**) Magnetization vs. Temperature curve under 100 Oe. (**d**) Stress-strain curve of Ni_54_Fe_19_Ga_27_ single crystal at a low strain rate of 0.001 s^−1^.

Figure 1(a) shows the room-temperature XRD patterns for Ni_54_Fe_19_Ga_27_ single crystal, as well as a powder sample for comparison. The polycrystalline powder sample has a cubic austenitic structure with main reflections of (220), (400) and (422) planes. For the single crystal sample, there observed only a single diffraction peak identified as the (420) diffraction for the *L*2_1_ structure. This indicates that the uniaxial stress is along the orientation of [420]_A_ of Ni_54_Fe_19_Ga_27_ single crystal. From the DSC curve in Fig. 1(b), MT with a hysteresis of about 5 K occurs around room temperature. The martensitic transformation start (*M*_s_) and finish (*M*_f_) temperatures, the austenite transformation start (*A*_s_) and finish (*A*_f_) temperatures, as well as the transformation enthalpies (∆*H*) were marked in Fig. 1(b). As shown in Fig. 1(c), the MT characteristic temperatures determined by magnetic measurement coincide to those in the calorimetry test, and the thermo-magnetization curve under the low field of 100 Oe indicates that the Curie temperature is about 315 K.

Entropy change ∆*S* can be evaluated by an expression as below:


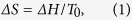


where *T*_0_ is the equilibrium temperature defined as (*M*_s_ + *A*_f_)/2^22^. For Ni_54_Fe_19_Ga_27_ single crystal, ∆*S* is calculated to be 15.9 J/Kg K with *T*_0_ = 281.7 K and ∆*H* = 4.5 J/g. The adiabatic temperature change related with the elastocaloric effect can be approximately estimated as:


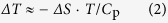


where *C*_p_ is the heat capacity and *T* is the ambient temperature during compression test. Considering the negligible influence of stress on *C*_p_, we take *C*_p_ as 29 J/mol K for both martensitic and austenite under constant uniaxial stress^23^. The resultant potential of maximum value of ∆*T* for Ni_54_Fe_19_Ga_27_ is about 9.8 K.

At isothermal condition, the stress-strain curve with a low strain rate of 0.001 s^−1^ is shown in Fig. 1(d). Upon loading, we can clearly see two successive transformation plateaus, which indicate the occurrence of the stress-induced martensitic transformation at 30 MPa followed by an inter-martensitic transformation at 40 MPa. The full transformation completes at 70 MPa with a transformation strain of 4.2%. According to the previous study by Sutou *et al.*^24^, the two-stage stress-induced MT can correspond to a sequence of *L*2_1_ − 14*M* − *L*1_0_ transformation. In the unloading process, a complete superelastic loop is obtained without residual strain. It should be noted that the *L*1_0_ martensite directly transforms back to *L*2_1_ austenite in the absence of inter-martensitic transformation.

With respect to the non-modulated *L*1_0_ martensite, the 14*M* martensite is featured by high density micro-twins with an ordered stacking structure^24^. The lattice misfit between 14*M* and *L*2_1_ structures is relatively smaller, which leads to a lower frictional resistance during phase boundary motion. Besides, as the de-twinning effect is hard to take place in 14*M* martensite^25^, the stress favors to create the undistorted habit plane of *L*2_1_/14*M* rather than that of *L*2_1_/*L*1_0_. Therefore, the 14*M* martensite is prefer to form by compressing along [420]_A_ axis, whereas the *L*1_0_ structure eventually stabilizes by further deformation. However, a direct *L*1_0_-*L*2_1_ transformation is likely to take place during unloading without the experience of the inter-martensitic transformation^25^. The difference in stress hysteresis, which is associated with energy dissipation, between martensitic and inter-martensitic transformations might change the reverse transition path compared to the forward transition^26^. This assumption has been validated in Ni-Mn-Ga single crystals^27^.

Superelastic hysteresis loop and associated elastocaloric effect kinetically rely on two parameters: (i) the strain rate that reflects the rate of heat transfer to environment, and (ii) the transformation strain that corresponds to the proportion of the transformed phase. Here, we quantify the effects of strain rate and transformation strain on superelastic properties and ∆*T*.

**Figure 2:**
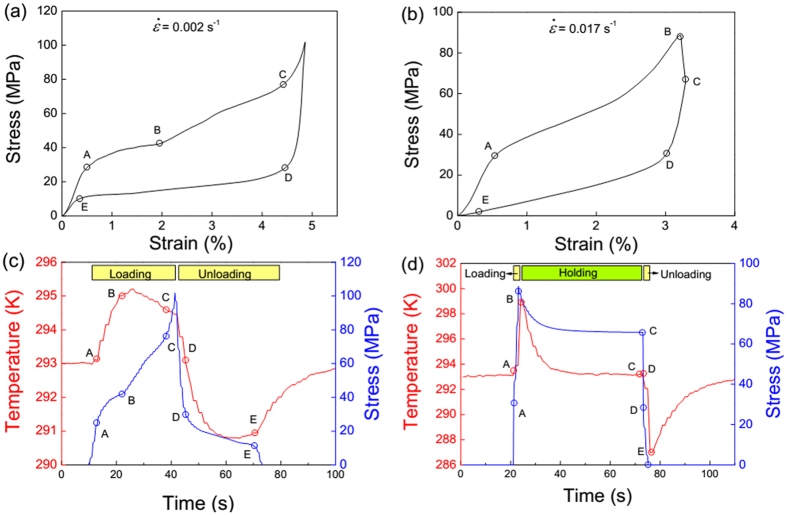
Stress-strain curves of Ni_54_Fe_19_Ga_27_ single crystal at the low strain rate of 0.002 s^−1^ without the stress-holding process (**a**) and the corresponding time dependence of temperature and stress variations (**c**), and at the high strain rate of 0.017 s^−1^ with the stress-holding step (**b**) and the corresponding time dependence of temperature and stress variations (**d**).

Figure 2 shows the stress-strain curves at the low strain rate of 0.002 s^−1^ without stress-holding process (a) and at the high strain rate of 0.017 s^−1^ with the holding step (b). The corresponding time dependence of temperature and stress variations at an initial temperature of 293 K are simultaneously recorded, as shown in Fig. 2(c,d). For the lower loading rate, the AB segment is the *L*2_1_ − 14*M* MT with a temperature raise of 2 K. In the BC segment indicating the 14*M* − *L*1_0_ inter-martensitic transformation, the temperature starts to slightly decrease. This is because the achieved large inter-transformation strain (2.5%) needs the longer loading time and therefore causes higher heat dissipation. Upon unloading the reverse MT starts at point D and finishes around point E, which gives rise to a cooling effect of about −2 K. Moreover, compared with the loading segments (AB and BC), the single segment (DE) in the stress-time response confirms the appearance of the direct *L*1_0_-*L*2_1_ transformation on unloading. In the case of the fast rate (0.017 s^−1^, Fig. 2b,d), the inter-martensitic transformation becomes smeared and a steeper temperature change can be observed on forward and reverse MTs. It is clear that the fast loading/unloading leads to a much higher temperature change about 7 K even with incomplete transformation, due to a better adiabatic condition. As the superelastic deformation is fully recovered, the ∆*T*-time response is nearly symmetric in loading/unloading cycle.

From the stress-strain curves under incremental loading at different strain rates (Fig. S1, Supplementary material S1), we found that with the increase of the transformation strain at a constant strain rate, the forward transformations follow nearly the same path but unloading protocols proceed with a low stress plateau, which widens the interval of loading and unloading curves. With the increased strain, the martensite transformation becomes fully completed and correspondingly the volume fraction of martensite is increased. Thus a lower stress plateau is observed in the reverse MT accompanying with the self-cooling effect. By increasing the strain rate from 0.003 to 0.025 s^−1^, the slopes of the transformation plateau increase and the stress hysteresis becomes larger, due to the significant self-heating and self-cooling effects at quasi-adiabatic conditions induced by the high strain rate. Besides, it should be noted that the superelastic loop cannot completely recover after unloading at high strain rate with large transformation strain. It is worth addressing that the unrecovered strain is not the consequence of austenitic plastic (permanent) deformation. It appears as the so-called “temporary residual strain (TRS)” phenomenon, which is usually encountered at high-strain-rate unloading due to its large stress hysteresis^28^. Such residual strains could lead to a relaxation processing and destroy adiabatic condition, thus result in a reduced ∆*T*.

**Figure 3:**
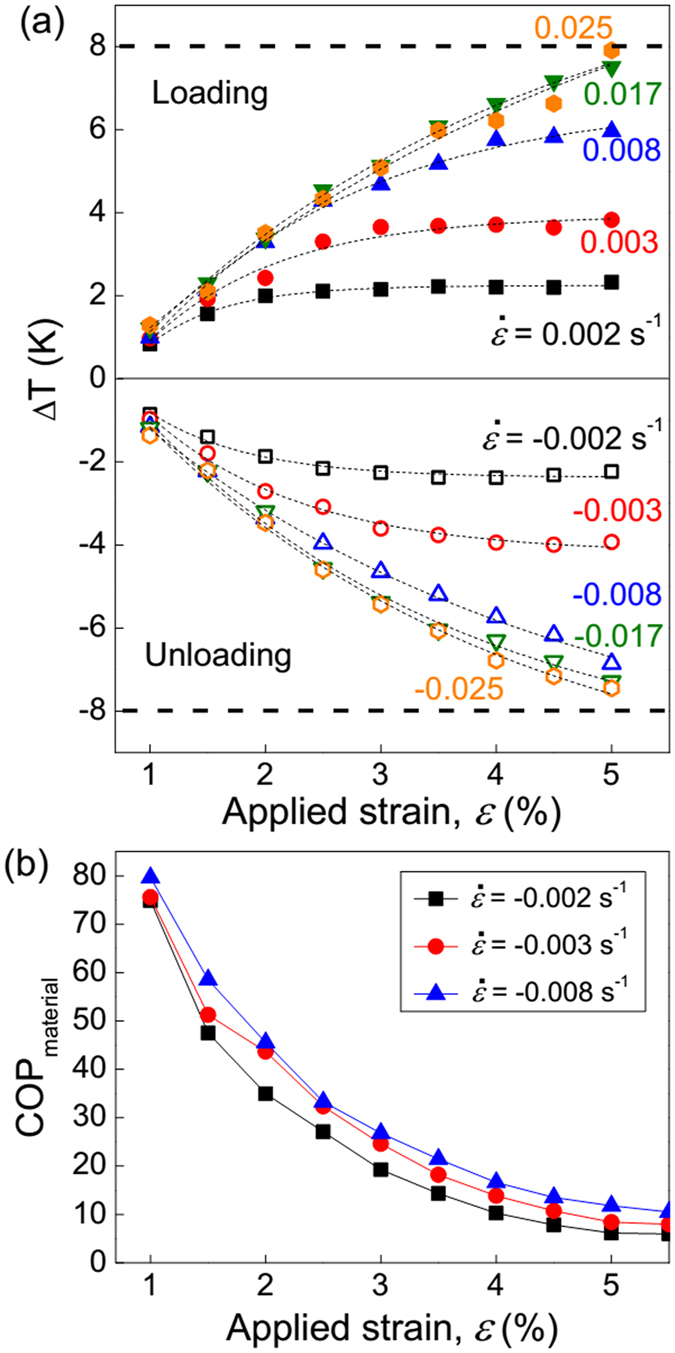
Temperature change ∆*T* vs. applied strain (*ε*) profiles with different strain rates (**a**) and calculated *COP*_material_ values in unloading protocol (**b**) at 293 K for Ni_54_Fe_19_Ga_27_ single crystal. Strain rates of 0.002, 0.003, 0.008, 0.017 and 0.025 s^−1^ are selected to perform the loading and unloading processes.

**Figure 4:**
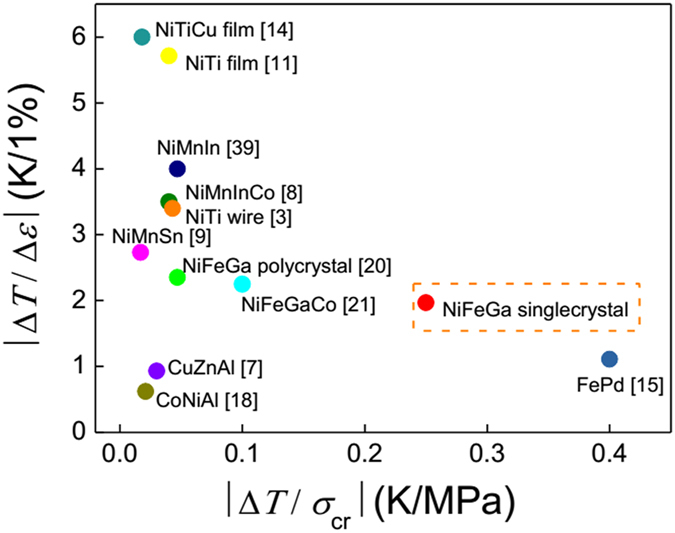
A comparison of the specific adiabatic temperature change (|∆*T*/*σ*_cr_| and |∆*T*/∆*ε*|) at room temperature for various elastocaloric materials. The data for Ni-Fe-Ga-Co single crystal was taken at 348 K. Except for the Ni-Fe-Ga single crystal, the data were taken from related literatures as quoted therein.

To explore the influence of superelastic properties on ∆*T*, the ∆*T* vs. applied strain (*ε*) diagram at different strain rates (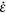) is plotted in Fig. 3(a). It can be seen that ∆*T* increases with *ε* and trends to be saturated eventually. Moreover, the measured ∆*T* exhibits an asymmetric behavior under high-strain rate loading/unloading. For example, the absolute value of ∆*T*_loading_ (8 K) is larger than that for ∆*T*_unloading_ (7.5 K) at a strain rate of 0.025 s^−1^. We ascribe this asymmetry as the result of the TRS effect. The maximum reversible elastocaloric temperature change (∆*T*_loading_ = ∆*T*_unloading_) is ±7.5 K at 3.8% transformation strain under 30 MPa driving force. The effective elastocaloric effects |∆*T*/*σ*_cr_| and |∆*T*/∆*ε*| are 0.25 K/MPa and 1.97 K/1%, respectively. Such specific mechanical cooling data are compared with those in other elastocaloric materials measured at room temperature (Fig. 4). For the cooling device, the small transformation stress represents a low input work and small strain implies a low noise. From that, one can notice that the Ni-Ti alloys exhibit a high ∆*T* under smaller strain but need high critical stress, while Ni-Mn-based samples are hard to be strained to a large value due to their intrinsic brittleness. The most significance for the present Ni-Fe-Ga single crystal is that the effective loading for elastocaloric cooling is rather low compared with existing martensitically transformed materials. Although the Fe-Pd alloy system requires even lower stress, its potential of ∆*T* is limited to 3 K owing to less latent heat releasing^15^.

**Figure 5:**
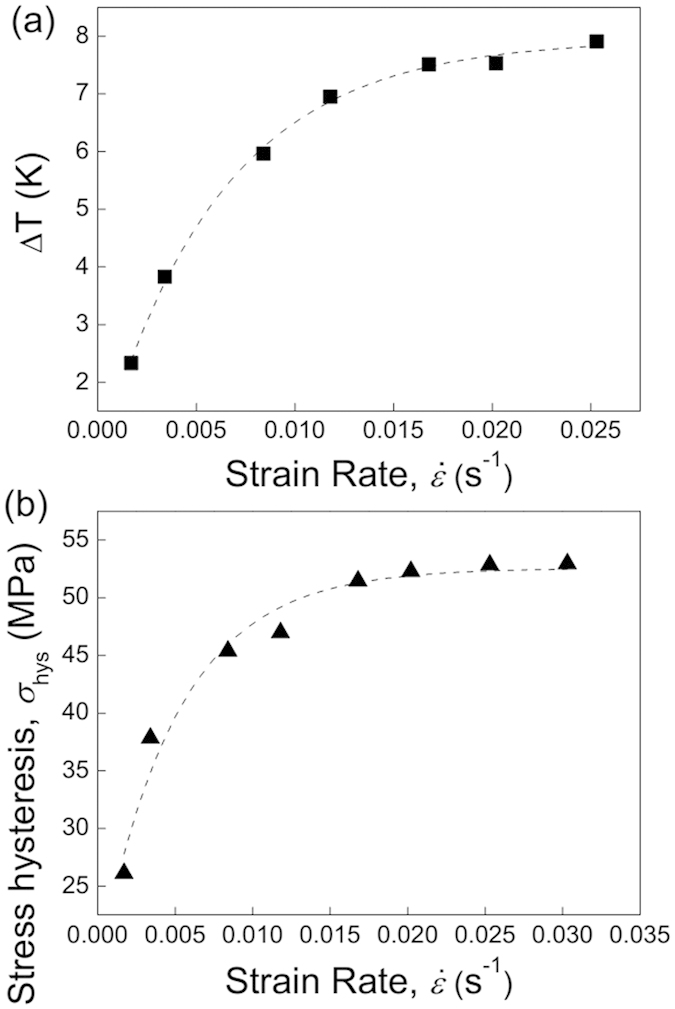
∆*T* (**a**) and stress hysteresis (**b**) as a function of strain rate by applying a constant strain of 5% at 293 K for Ni_54_Fe_19_Ga_27_ single crystal.

Figure 5(a) shows strain rate dependence of ∆*T* by keeping a constant applied strain of 5%. Similar to the tendency of ∆*T* vs. *ε* in Fig. 3(a), the value of ∆*T* drastically increases with high strain rate, and has close magnitude when 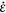 above 0.017 s^−1^. These results suggest that solely increasing either strain rate or transformation strain has limited impact on the enhancement of ∆*T*. On the contrary, the adoption of very high values of *ε* and 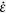 usually brings about some unwanted effects for elastocaloric refrigeration, such as the asymmetric ∆*T* distribution and irreversible energy losses. In order to extend the fatigue life, the optimization of the applied strain rate and strain magnitude requires to be considered in elastocaloric cooling prototype^28^,^29^. Therefore, we discuss the optimal MT strain range for balancing a high coefficient of performance (*COP*) and large ∆*T*.

According to reported work by Ossmer *et al.*^11^, the *COP* of elastocaloric materials is defined by dividing the cooling power (∆*Q*) by input work (∆*W*):


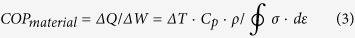


where *σ* is the applied stress and *ρ* is the density of Ni_54_Fe_19_Ga_27_ (8.58 × 10^3^ kg/m^3^). The adiabatic temperature change ∆*T* is obtained in the unloading process, and the input work is associated with the enclosed area of stress-strain curves. Fig. 3(b) shows calculated values of *COP*_material_ as a function of applied strain at different strain rates. With increasing the applied strain, the *COP*_material_ trends to decrease while ∆*T* is increased. A large transformed volume leads to a high temperature change of specimen. But the disadvantage of the high strain for fully transforming is the increase of stress hysteresis, which reduces the cooling efficiency and fatigue life. Meanwhile, ∆*T* does not increase further with strain once a complete MT is reached. The optimized region (2–3% transformation strain) might be regarded as the balance of *COP*_material_ and ∆*T*. In this region, the elastocaloric material owns an appropriate ∆*T* without damage the cooling efficient and fatigue life. It should be noted that *COP*_material_ is different from *COP* for the integrated cooling system. *COP*_material_ measures the energy conversion efficiency from the intrinsic material properties only, by assuming that the materials undergo the cooling cycle with ideal system configuration^30^. It is a material performance metric to compare different elastocaloric materials. However, the input work of *COP*_system_ contains extra irreversible losses portions, such as pump work, motor mechanical losses and heat transfer losses in a real cooling cycle system. ∆*T* of *COP*_system_ is defined as the temperature change integrated during the entire period of the heat transfer between the material and its ambient^29^. Therefore, *COP*_system_ has much smaller values than *COP*_material_^31^.

From above, we know that the maximum value of ∆*T* strongly depends on the applied strain and strain rate. In fact, the ∆*T* vs. time profile is also drastically influenced by the characteristics of stress induced MT.

**Figure 6:**
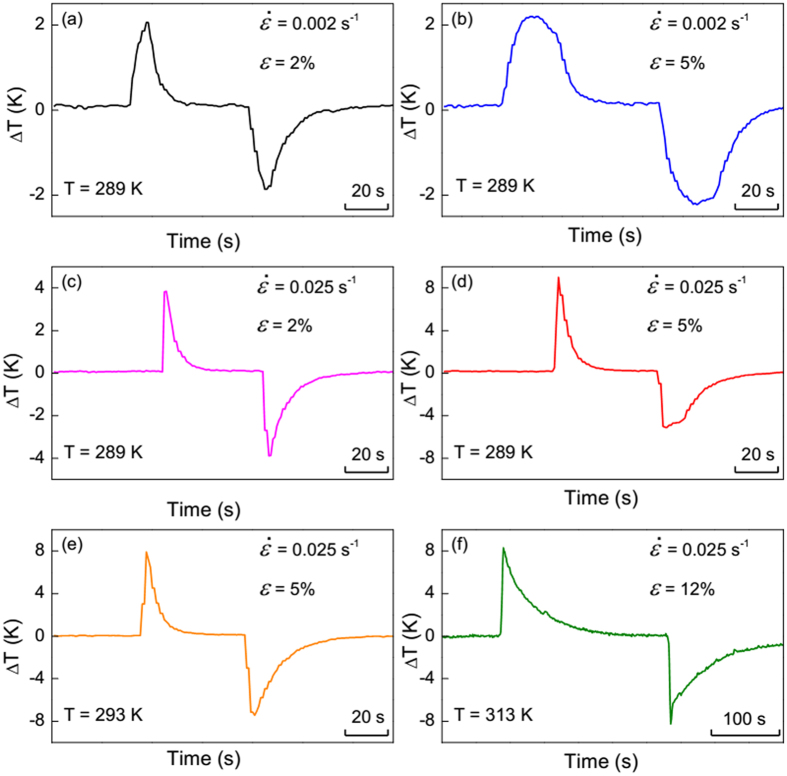
∆*T* vs. time profiles at selected strain rates, transformation strains and starting temperatures for Ni_54_Fe_19_Ga_27_ single crystal.

The ∆*T* vs. time profiles at selected transformation strains, strain rates and ambient temperatures for Ni_54_Fe_19_Ga_27_ single crystal are shown in Fig. 6. At a low strain rate of 0.002 s^−1^ (Fig. 6(a,b)) at 289 K, ∆*T* curves obtained in loading and unloading process are almost symmetric and exhibit equivalent maximum peak values (about ±2 K). The difference is that one can see a broader peak at a larger strain (*ε* = 5%). This result reflects that the specimen needs longer time to reach the larger strain, during which the heat losses somehow counteract the latent heat.

The temperature rises up to a higher value on fast loading at 0.025 s^−1^ (Fig. 6(c,d)). It reaches +4 K for *ε* = 2% and +8 K for *ε* = 5% upon loading. However, we have observed a significant asymmetry of ∆*T* vs. time profile in Fig. 6(d) owing to the appearance of TRS phenomenon, which is considered as an anelastic behavior in Ni_54_Fe_19_Ga_27_ elastocaloric material. Upon fast unloading, TRS cannot resume immediately, but can be gradually recovered after a few minutes, representing a typical anelastic behavior. The anelasticity is different from the conventional mechanical properties showing either elasticity (resume its original shape immediately once the external stress is removed) or plasticity (irreversible residual strain associated with the dislocation slip mechanism in ductile materials)^32^. The anelasticity is evidenced by a delay in shape recovery after the mechanical stress releases. In this case, the strain response falls behind the stress application. Such a relaxation process is probably due to the large elastocaloric effect at fast unloading. After unloading, as the temperature slowly rises to the ambient, the residual martensite is able to transform back to austenite and shape recovers. The TRS effect is directly related to the reversibility of ∆*T* and the cooling capacity of eCE materials.

Since the austenite is more stable at higher temperatures, a larger critical stress (*σ*_cr_) is applied to drive MT at elevated testing temperature. In this case, the superelastic loops might be completed even by fast deformation. This leads to the vanishing of TRS and therefore the re-attained symmetric ∆*T* profile, as shown in Fig. 6(e,f). Also, one can observe the same ∆*T* magnitude of about 8 K at 293 and 313 K, indicating a large temperature window at least 25 K in our Ni_54_Fe_19_Ga_27_ single crystal. Assuming that the σ_cr_ can be as high as 1000 MPa in high-temperature Ni-Fe-Ga-based shape memory alloys^33^ and the σ_cr_ is only 106 MPa at 313 K in present study, a further broader temperature window is expected in our sample. One should notice that at 313 K the specimen takes much longer relaxation time (about 150 s) to return to its initial temperature during holding process, which is ascribed to the establishment of better adiabatic conditions by reducing air convention in the heating furnace.

The stress hysteresis is primarily influenced by frictional resistance and variant interaction^16^. The irreversible frictional dissipation of the interface between austenite and martensite promotes higher stress hysteresis. Likewise, the interaction between existing variants and a nucleating one in multi-variant martensitic transformation contributes an additive part to the increase of the stress hysteresis. We further analyze the impact of strain, strain rate and temperature change ∆*T* on the stress hysteresis (*σ*_hys_, defined as the interval between the onsets of stress-induced forward and reverse MTs).

From the stress hysteresis as a function of the strain rate Fig. 5(b), we found that when 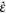 is above 0.017 s^−1^, the stress hysteresis is stabilized as about 50 MPa. There are two mechanisms to interpret this phenomenon. First, the strain-rate dependent stress hysteresis is generally influenced by the efficiency of heat transfer between the specimen and its environment^34^. For the high strain rate upon loading/unloading, the latent heat cannot be efficiently released/absorbed due to the limited heat transfer, and therefore self-heating/cooling effect occurs^35^. The significant local temperature change of the sample requires the drastic increase/decrease of applied stress to drive the forward and reverse MTs. Therefore the overall size of hysteresis grows. The second reason responsible for the increased stress hysteresis is related to the mobility of interface movement at higher strain rate. In this case, the interface moves quickly after the first creation of the nucleus, and there is little time for the stress relaxation in the fronts^36^. Thus, the internal friction resistance against the interface movement becomes large and the stress level to drive the martensitic nucleation and interface propagation increases, which leads to a wider hysteresis interval.

**Figure 7:**
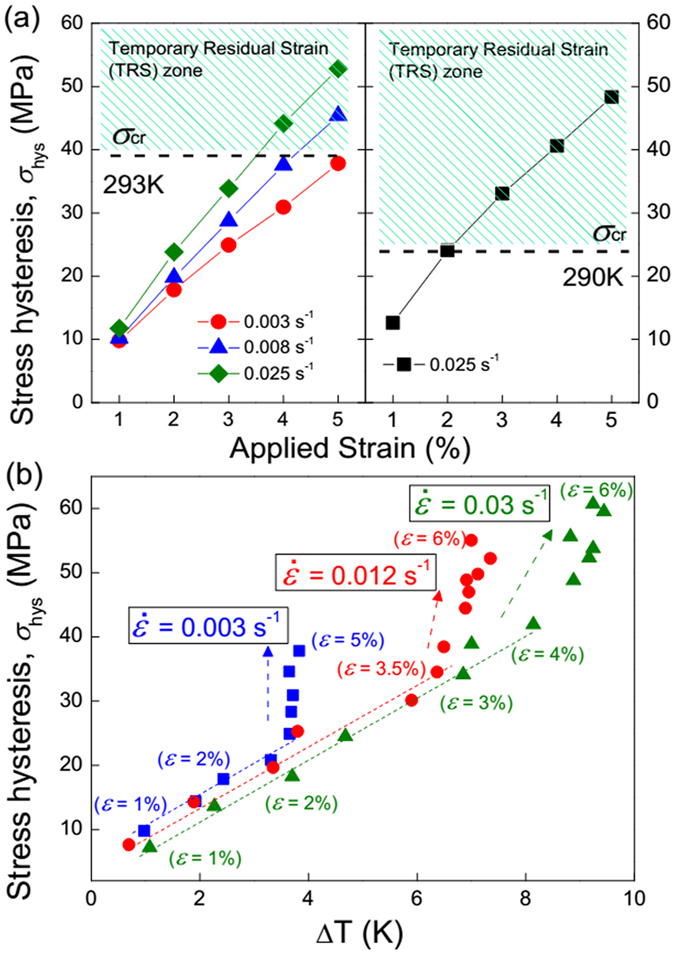
(**a**) The applied strain dependence of stress hysteresis at selected strain rates at 293 K and 290 K. The dash lines indicate the value of critical stress (*σ*_cr_). The hatched green area indicates the appearance of temporary residual strain. (**b**) Stress hysteresis vs. ∆*T* profiles for Ni_54_Fe_19_Ga_27_ single crystal in different deformation conditions at 293 K. Stress hysteresis as a function of ∆*T* under incremental strain from 1% to 5% by a step of 0.5% at 0.003 s^−1^. Stress hysteresis as a function of ∆*T* under incremental strain from 1% to 6% by a step of 0.5% at 0.012 and 0.03 s^−1^. The dash lines and arrows indicate the variation tendency of *σ*_hys_ vs. ∆*T*.

In Fig. 7(a), the stress hysteresis linearly increases with increased applied strain for each strain rate. The slope of *σ*_hys_ over *ε*, however, is larger at the higher strain rate. By comparing the magnitude of *σ*_hys_ and *σ*_cr_ at different strain rates at a constant initial temperature, a criterion to judge the reversibility of dynamic superelastic behavior and associated temperature change can be obtained, as described in follows. When *σ*_hys_ is lower than *σ*_cr_, the superelastic deformation is highly recoverable, and then a symmetric ∆*T* profile can be obtained. In case that *σ*_hys_ is larger than *σ*_cr_ (as marked in the hatched green area), the corresponding strain cannot be recovered immediately when the applied stress is removed, which indicates the appearance of TRS effect. This gives rise to an asymmetry ∆*T* in response to loading/unloading cycles. Furthermore, the testing temperature should be taken into account the above proposed criterion, with which the critical stress (*σ*_cr_) almost linearly increases. For instance, the sample strained up to 3% at 0.025 s^−1^ exhibits a pronounced TRS at 290 K, but undergoes a reversible stress-induced transition and highly symmetric temperature change at higher testing temperature of 293 K.

From above, we have known that ∆*T* is closely interlinked with 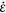 and *ε*, and these three parameters decide the overall stress hysteresis. The question arises that which factor is dominating in a specific condition. To solve this problem, we plot a comprehensive diagram to further illustrate the impact factors of hysteresis, as shown in Fig. 7(b). For the lower value of 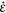 of 0.003 s^−1^, *σ*_hys_ increases with both ∆*T* and *ε* (<3%), and continues to increase with the *ε* (>3%) but get to be independent of ∆*T*. This indicates that the stress hysteresis mainly relies on the thermal effect at lower strain, while depends on the transformed volume fraction at higher strain range. For the higher strain rates 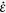 of 0.012 and 0.03 s^−1^, there still observed an evident inflexion in the curves of *σ*_hys_ vs. ∆*T*. However, due to the better adiabatic condition, ∆*T* trends to slightly increase at large strains after the inflexion. Therefore, the combined effect of temperature fluctuation and transition proportion causes the increased *σ*_hys_ for the high 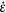 and *ε*.

**Figure 8:**
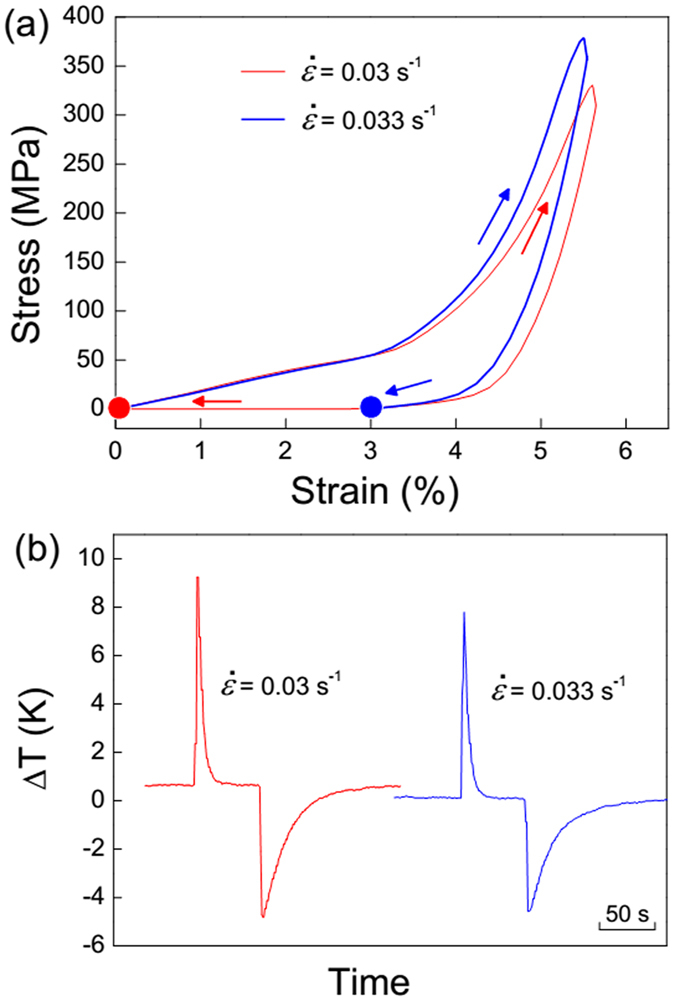
Stress vs. strain responses (**a**) and ∆*T* vs. time profiles (**b**) for Ni_54_Fe_19_Ga_27_ single crystal under quasi-adiabatic compression conditions with high strain rates of 0.03 and 0.033 s^−1^ at 293 K.

Aforementioned results conclude that solely increasing strain rate does not always benefit the large temperature change. Figure 8 shows stress vs. strain and ∆*T* vs. time profiles under quasi-adiabatic compression conditions at high strain rates of 0.03 and 0.033 s^−1^. For both high-rate deformations, there retained a certain of residual strain after unloading. The difference is that the residual strain at 0.03 s^−1^ can be recovered after a while (TRS effect), but the sample was plastically deformed at 0.033 s^−1^. When the strain rate increases from 0.03 to 0.033 s^−1^, ∆*T*_loading_ obviously declines from 9.4 to 7.8 K. The residual martensite at 0.033 s^−1^ loading/unloading was characterized by TEM observation (Fig. S2). The martensitic variants exhibits twisted morphology and the interface becomes ambiguous due to severe plastic deformation. The corresponding selected area electron diffraction (SAED) indicates that the martensite can be indexed to be a modulated type with four-layered orthorhombic structure (4*O*)^37^,^38^.

Finally, we exploit the potential of ∆*T* in Ni_54_Fe_19_Ga_27_ single crystals. The isothermal entropy change is estimated using Clausius-Clapeyron equation^39^:


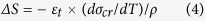


where *ε*_*t*_ is the transformation strain of Ni_54_Fe_19_Ga_27_ specimen and d*σ*_cr_/*dT* is the working temperature dependent critical transformation stress that is 3.3 MPa/K. Using Eq. (4), the estimated isothermal entropy change is 16.2 J/kgK for loading. By using Eq. (2), the theoretically maximum value of ∆*T* is calculated to be 10 K. This value is in a good agreement with that obtained by DSC measurement (9.8 K). Experimentally, with the optimal input parameters of the strain rate of 0.03 s^−1^ and strain level of about 5.5%, we have achieved the maximum value of ∆*T* (9.4 K), which is very approaching the upper bound of ∆*T*. For the further consideration, in order to realize the reversible temperature change, the stress hysteresis needs to be reduced and a slightly higher starting environmental temperature is desirable.

In summary, the superelastic behavior and the related elastocaloric properties of the Ni_54_Fe_19_Ga_27_ single crystal under different dynamic deformation conditions have been investigated. Both the applied strain and strain rate strongly impact elastocaloric effect. A reversible adiabatic ∆*T* of ±7.5 K was achieved. As a rather small hysteresis heat was estimated to be 0.3 K, the symmetric of temperature change profile was not obviously influenced by dissipative heat. The measured maximum ∆*T* is 9.4 K upon loading with 0.03 s^−1^ at 293 K, while the plastic deformation of martensite occurs at a very high strain rate of 0.033 s^−1^. ∆*T* increases with transformation strain and strain rate and finally trends to be saturated. It is observed that solely increasing either strain magnitude or strain rate cannot promote the cooling capacity, but leads to the asymmetric ∆*T* distribution and irreversible energy losses. We have established a criterion to judge the reversibility of dynamic superelastic behavior and associated temperature change. The stress hysteresis mainly relies on the thermal effect at lower strain, while depends on the transformed volume fraction at higher strain range.

## Methods

### Sample preparation and characterization

A Ni_54_Fe_19_Ga_27_ (at.%) rod alloy with the diameter of 7 mm as the mast alloy was prepared by suction casting method in argon atmosphere from high pure Ni (99.99%), Fe (99.99%) and Ga (99.99%). Single crystal was grown by float-zone-melting technique in a double ellipsoid furnace (Quantum Design IR image furnace G2) filled with 99.99% argon gas pressure of 0.8 MPa. The focused zone of the rod was heated and then formed a stable molten zone of ~10 mm in height. The high-quality crystal with less composition segregation was grown using a pulling rate of 8 mm/h and rotating rate of 8 rpm. A rectangular sample in size of 5 mm × 5 mm × 10 mm with the long axis along the [420]_A_-direction (A represents the parent austenite phase) for compressive tests was cut by electro-discharge machining from the single crystal. MT temperatures and latent heat were measured by a Pyris Diamond differential scanning calorimeter (DSC) with a heating/cooling rate 10 K/min. A superconducting quantum interference device (SQUID) magnetometer Quantum Design MPMS was used to confirm MT temperatures and analysis magnetic properties. The crystal structure of the single crystal was determined by X-ray diffraction (XRD) using Cu-Kα radiation. Microstructure of the specimen subjected to the fast loading was observed in a transmission electron microscope (JOEL 2100 HR TEM).

### Measurements of elastocaloric effect

Compression experiments were conducted on a universal testing machine (SUN UTM5000). The temperature change (∆*T*) of the specimen induced by the stress-induced martensite transformation was monitored by a K-type thermocouple pasted on the specimen surface center. The stress-strain curves under different strain rates were recorded at 289, 293 and 313 K. The sample was deformed in a heating furnace at 313 K. The strain rate 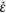 varies from low to high speeds: 0.001 s^−1^, 0.002 s^−1^, 0.003 s^−1^, 0.008 s^−1^, 0.012 s^−1^, 0.017 s^−1^, 0.025 s^−1^, 0.03 s^−1^ and 0.033 s^−1^. It should be noted that for the low-dimensioned Ni-Ti film^11^, an even higher unloading rate (1 s^−1^) was needed to avoid heat dissipation. In the case of our bulk samples, the near adiabatic condition was relatively easily approached by applying lower strain rate (e.g. 0.025 s^−1^). The temperature changes under compression test with different strain rates at different temperatures were measured in two processes. The first process is the stress-driven mode. The rectangular sample was loaded at a strain rate until reaching to the target stress and then unloaded at the same rate without a stress-holding step. The second process was performed with stain-driven mode. The specimen was loaded up to the target strain and hold for 50 s to ensure that the sample temperature fell back to the initial value. Afterwards, the specimen was unloaded at the same strain rate. The holding process reflected the thermal conduction or emission with environments, which is analogous to the heat-exchange step between the elastocaloric material and fluid in refrigeration cycles.

## Additional Information

**How to cite this article**: Li, Y. *et al.* Giant and reversible room-temperature elastocaloric effect in a single-crystalline Ni-Fe-Ga magnetic shape memory alloy. *Sci. Rep.* **6**, 25500; doi: 10.1038/srep25500 (2016).

## Supplementary Material

Supplementary Information
