# Supplementary material for: Giant and reversible room-temperature elastocaloric effect in a single-crystalline Ni-Fe-Ga magnetic shape memory alloy
Source: Sci Rep. 2016 May 3;6:25500. doi: 10.1038/srep25500 (PMC4853795; doi:10.1038/srep25500)
Supplement: Supplementary Information [file srep25500-s1.pdf]

SUPPLEMENTAL MATERIAL

Giant and reversible room-temperature elastocaloric effect in a single-crystalline Ni-Fe-Ga magnetic shape memory alloy

Yang Li[a,b,c], Dewei Zhao[a,b] and Jian Liu[a,b*]

[a] Key Laboratory of Magnetic Materials and Devices, Ningbo Institute of Material Technology and Engineering, CAS, Ningbo 315201, China

[b] Zhejiang Province Key Laboratory of Magnetic Materials and Application Technology, Ningbo Institute of Material Technology and Engineering, CAS, Ningbo 315201, China

[c] School of Materials Science and Engineering, Shanghai University, Shanghai 200072, China

[*] Corresponding author: liujian@nimte.ac.cn

Fig. S1 shows the stress-strain curves of the $Ni_{54}Fe_{19}Ga_{27}$ single crystal under incremental loading at different strain rates. With the increase of the transformation strain at a constant strain rate, the forward transformations follow nearly the same path but unloading protocols proceed with a low stress plateau, which widens the interval of loading and unloading curves. By increasing the strain rate, the slope of the transformation plateau increases and the stress hysteresis becomes larger. The superelastic loop cannot completely recover after unloading at high strain rate with large transformation strain. The residual strains could lead to a relaxation processing and destroy adiabatic condition, thus result in a reduced ΔT.

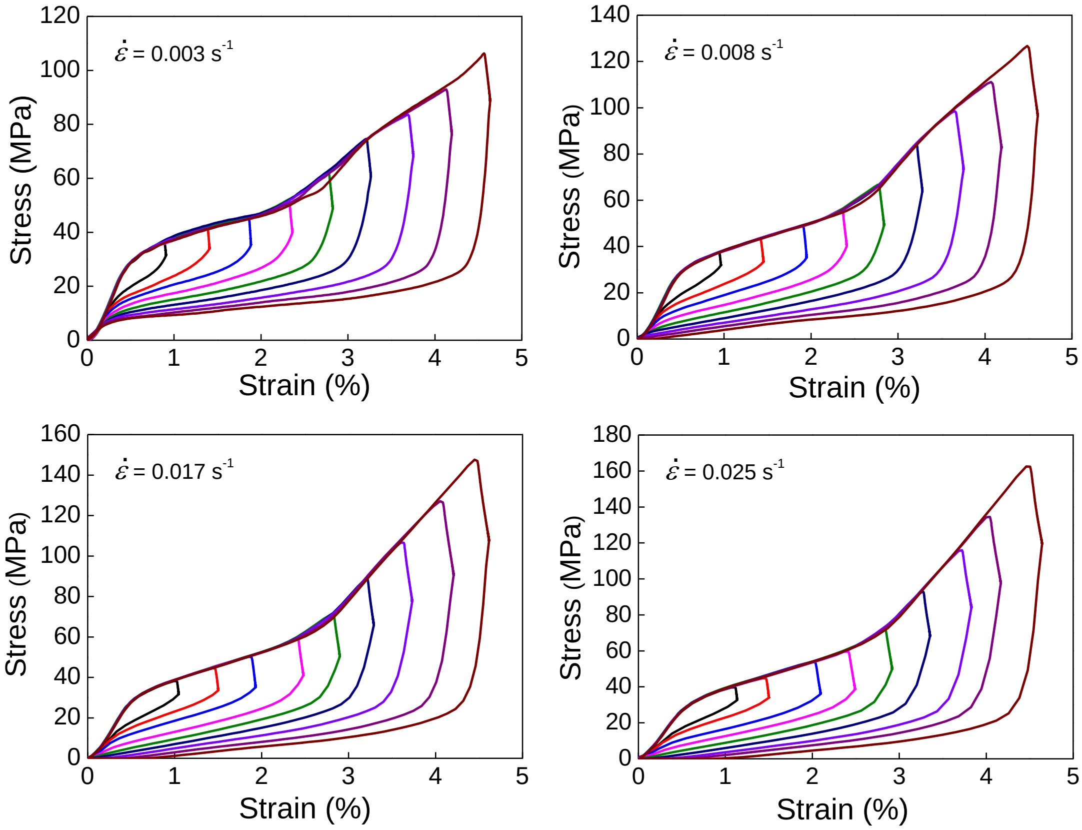

Fig. S1. Stress-strain curves of $Ni_{54}Fe_{19}Ga_{27}$ single crystal under incremental loading with different strain rates at an initial temperature of 293 K.

Fig. S2 shows the residual martensite on loading/unloading at ±0.033 s^{-1} was characterized by TEM observation. The martensitic variants exhibits twisted morphology and the interface becomes ambiguous due to severe plastic deformation. The corresponding selected area electron diffraction (SAED) indicates that the martensite can be indexed to be a modulated type with four-layered orthorhombic structure (4*O*)

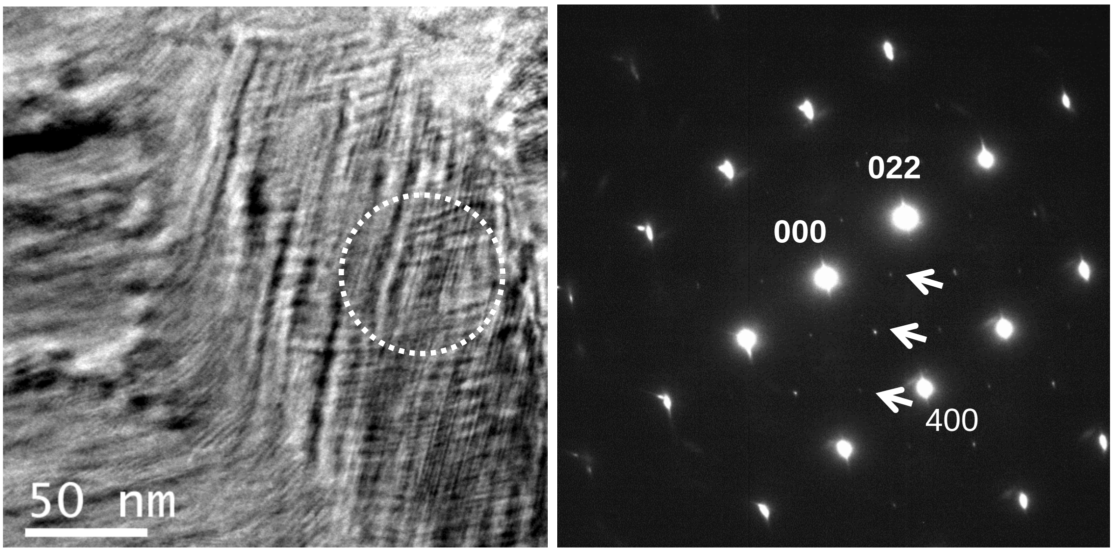

Fig. S2. TEM bright field image of $Ni_{54}Fe_{19}Ga_{27}$ single crystal after loading and unloading with 0.033 s^{-1} at room temperature (left) and the corresponding selected area electron diffraction (SAED) taken from the areas marked by white cycle in (right).
